# Transcription analyses of differentially expressed mRNAs, lncRNAs, circRNAs, and miRNAs in the growth plate of rats with glucocorticoid-induced growth retardation

**DOI:** 10.7717/peerj.14603

**Published:** 2023-01-16

**Authors:** Mingyue Yin, Junqi Wang, Juanjuan Zhang, Wei Wang, Wenli Lu, Fei Xu, Xiaoyu Ma, Sheng Lyu, Lifen Chen, Lidan Zhang, Zhiya Dong, Yuan Xiao

**Affiliations:** 1 Department of Pediatrics, Ruijin Hospital, Shanghai Jiao Tong University, School of Medicine, Shanghai, China; 2 Department of Pediatrics, Liqun Hospital, Putuo District, Shanghai, China

**Keywords:** Glucocorticoid, Growth retardation, Growth plate, RNA sequencing, Competing endogenous RNA, Non-coding RNA

## Abstract

**Background:**

Glucocorticoids (GCs) are commonly used to treat autoimmune diseases and malignancies in children and adolescents. Growth retardation is a common adverse effect of GC treatment in pediatric patients. Accumulating evidence indicates that non-coding RNAs (ncRNAs) are involved in the pathogenesis of glucocorticoid-induced growth retardation (GIGR), but the roles of specific ncRNAs in growth remain largely unknown.

**Methods:**

In this study, 2-week-old male Sprague-Dawley rats had been treated with 2 mg/kg/d of dexamethasone for 7 or 14 days, after which the growth plate tissues were collected for high-throughput RNA sequencing to identify differentially expressed mRNAs, lncRNAs, circRNAs, and miRNAs in GIGR rats.

**Results:**

Transcriptomic analysis identified 1,718 mRNAs, 896 lncRNAs, 60 circRNAs, and 72 miRNAs with different expression levels in the 7d group. In the 14d group, 1,515 mRNAs, 880 lncRNAs, 46 circRNAs, and 55 miRNAs with differential expression were identified. Four mRNAs and four miRNAs that may be closely associated with the development of GIGR were further validated by real-time quantitative fluorescence PCR. Function enrichment analysis indicated that the PI3K-Akt signaling pathway, NF-kappa B signaling pathway, and TGF-*β* signaling pathway participated in the development of the GIGR. Moreover, the constructed ceRNA networks suggested that several miRNAs (including miR-140-3p and miR-127-3p) might play an important role in the pathogenesis of GIGR.

**Conclusions:**

These results provide new insights and important clues for exploring the molecular mechanisms underlying GIGR.

## Introduction

Glucocorticoid-induced growth retardation (GIGR) is a common side effect associated with long-term glucocorticoid (GC) treatment in children (Grigorescu-Sido et al., 2003; Ma et al., 2019). This treatment prevents many pediatric patients from reaching their expected target heights in adulthood, dramatically impacting their quality of life and psychosocial health. After discontinuation of glucocorticoid therapy, although a certain degree of catch-up growth would occur in some children (Browne et al., 2018), their adult height remained inevitably compromised (Kelly et al., 2012; Ribeiro et al., 2015). Furthermore, there are no therapies recognized as safe and effective in clinical practice for the prevention of GIGR. So, GIGR is one of the major concerns when children receive GC treatment.

The growth plate is a thin layer of cartilage located at the metaphysis that is essential for endochondral ossification (Kronenberg, 2003). Direct and indirect effects of GCs on the growth plate and its cartilage components are considered to be the source of their impact on growth. GCs can reduce the height of the epiphyseal growth plate, affect chondrocyte differentiation, inhibit chondrocyte proliferation, and induce chondrocyte apoptosis (Chrysis, Ritzen & Sävendahl, 2003; Hartmann et al., 2016; Zaman et al., 2019). Together, these effects lead to inhibition of longitudinal bone growth. However, the mechanisms behind these effects remain elusive.

An integrated analysis of protein-encoding genes as well as noncoding RNAs (ncRNAs) may reveal the underlying mechanism of GIGR. With the rapid development of high-throughput sequencing technology, many ncRNAs that were previously considered nonfunctional, including long non-coding RNAs (lncRNAs), circular RNAs (circRNAs), and microRNAs (miRNAs), have been identified and proven to play vital roles in various physiological and pathological processes, including endochondral ossification (Anastasiadou, Jacob & Slack, 2018; Razmara et al., 2019; Zhao et al., 2018). Li et al. (2014) used microarray analysis to analyze the expression profiles of miRNAs in human bone marrow-derived mesenchymal stem cells (hBMSCs) induced by dexamethasone (Dex) (10^−6^/ L; 12d) and found that some miRNAs, including miR-155, miR-199a-5p, miR-21, and miR-140-3p correlated with osteogenic differentiation and bone formation. Liu et al. (2018) found that the lncRNA Gas5 and miR-21 are involved in chondrocyte proliferation and apoptosis induced by GC *in vivo* and *in vitro*. In addition, Hao et al. (2021) observed that overexpression of CircPVT1 significantly alleviated GC-induced apoptosis and proliferation inhibition of hBMSCS cells. Therefore, it can be speculated that GC may induce growth retardation by affecting ncRNA and mRNA expression in the growth plate.

However, a knowledge gap currently exists regarding the effect of GC on ncRNA and mRNA expression in the growth plate. In this study, we attempted to reveal the changes of ncRNA and mRNA in the growth plate tissues by high-throughput RNA sequencing and bioinformatics analysis to provide a theoretical basis for elucidating the mechanism of GIGR. Gene Ontology (GO) analysis, Kyoto Encyclopedia of Genes and Genomes (KEGG) pathway analysis, and Gene Set Enrichment Analysis (GSEA) were performed to detect the potential functions and pathways of differentially expressed genes. We also reported the ceRNA networks involved in GIGR, which provides a new perspective for clarifying the gene expression regulatory network constructed by transcriptome and adds more dimensions for analyzing the molecular mechanism of GIGR.

## Materials & Methods

### Reagents

5 mg/mL of pharmaceutical-grade dexamethasone (Dex) sodium phosphate (Chenxin Pharmaceutical Co., Qidong, China) was diluted with 0.9% saline and administered intraperitoneally (i.p.) at a dose of 2.0 mg/kg/d in experimental rats in accord with a previous study (Liu et al., 2018), which has been shown to induce growth retardation. Control rats received the same volume of saline.

### GIGR rat model

The Institutional Animal Care and Use Committee of Ruijin Hospital, Shanghai Jiao Tong University School of Medicine approved these studies. The animal handling methods and procedures used in this study complied with the guidelines of the Institutional Animal Care and Use Committee. All methods were carried out in accordance with ARRIVE guidelines. SPF-level pregnant Sprague-Dawley (SD) rats were obtained from Vital River Laboratory (Beijing, China). Then newborn male SD rats were randomly assigned to cages and housed in a controlled environment (under controlled temperature, with a 12 h/12 h light dark cycle, with food and water provided *ad libitum*). Because the pregnant rats did not give birth on the same day. We performed a total of three batches of experiments and each batch rats were randomly divided into four groups. In short, 32 rats (2 weeks old, weight 33.79 ± 3.18 g) were randomly divided into four groups: (1) a Dex 7d group (2 mg/kg/d Dex for 7d, i.p., *n* = 9), (2) a Control 7d group (saline for 7d, i.p., *n* = 7), (3) a Dex 14d group (2 mg/kg/d Dex for 14d, i.p., *n* = 10), and (4) a Control 14d group (saline for 14 d, i.p., *n* = 6).

### Evaluation of growth

Rats were weighed first, then injected with saline or Dex at 8:00 am daily. Naso-anal length was measured under anesthesia once per week. After 7 or 14 days of injection, bilateral femurs and tibias were dissected and separated, and lengths were measured with a Vernier caliper.

### Samples collection

The day after the final Dex or saline administration, rats were deeply anesthetized with pentobarbital sodium (40 mg/kg, i.p.). All anaesthetised animals were euthanized by cardiac blood collection after loss of consciousness. Then the right proximal tibial growth plate tissues were dissected and separated, and snap-frozen immediately in liquid nitrogen.

### Quantitative histology of the growth plate

At the end of the 7- or 14-day injection period, the left proximal tibial tissue of rats was collected. Tissues were fixed in 4% paraformaldehyde for 24 h, decalcified in 0.5 M EDTA (Servicebio, Wuhan, China), embedded in paraffin, sectioned, and stained with hematoxylin and eosin (HE). These sections were then microphotographed with an optical microscope to observe morphological changes in the rats’ growth plates. The height of each zone of the growth plate was measured based on the distinct morphological characteristics of the different growth plate zones. At least 15 independent histological measurements were taken within the central two-thirds of the growth plate using Image J software (National Institutes of Health (NIH), Bethesda, MD, United States). The resting zone (R zone) was defined as single scattered cartilage cells. The proliferative zone (P zone) was defined as a region of flat chondrocytes with columnar displacement. The hypertrophic zone (H zone) is located below the P zone where the cells have significantly increased in size (increase by 5–10 times) (Su et al., 2021).

### Assessment of apoptosis and proliferation in growth plates

Terminal deoxynucleotidyl transferase dUTP nick end labeling (TUNEL) was used to analyze apoptosis in growth plates using a TUNEL Kit (Roche, Basel, Switzerland). TUNEL-positive cells (apoptotic cells) were counted within three randomly chosen fields in the H zone of each growth plate and expressed as the percentage of positive cells per field. A rabbit anti-proliferating cell nuclear antigen (PCNA) monoclonal antibody (1:500, ab92552; Abcam, Cambridge, UK) was used to assess proliferation in the P zone of the rat growth plate in three randomly chosen fields, and positive cells were also expressed as the percentage of positive cells per field. The calculation of the positive cells in each growth plate was based on at least 100 cells. We randomly selected three to four rats from each group dedicated to this experiment. The investigator conducting assessment did not know which treatment each sample had received.

### RNA extraction

Total RNA was extracted from each sample using TRIzol reagent (Invitrogen, Waltham, MA, USA). The degradation and contamination of total RNA were examined by 1% agarose gel electrophoresis. And the purity and concentration of the extracted RNA were evaluated using NanoPhotometer^^®^^ spectrophotometer (IMPLEN, Westlake Village, CA, USA). RNA integrity and quantity were assessed using the RNA Nano 6000 Assay Kit of the Bioanalyzer 2100 system (Agilent Technologies, Santa Clara, CA, USA). The total RNA of all samples had RNA integrity values greater than seven, which means that they are suitable for constructing sequencing libraries. We randomly selected three samples from each group for subsequent high-throughput sequencing.

### Library construction and high-throughput sequencing

We established two libraries: the chain-specific library and the small RNA library. For lncRNA, mRNA and circRNA sequencing, the chain-specific library was constructed using the TruSeq Stranded Total RNA Library Prep Plant/Gold/Globin +NEBNext^^®^^ Ultra Directional RNA Library Prep Kit (Illumina, San Diego, CA, USA) in accordance with the manufacturer’s instructions. Then the chain-specific library was subjected to PE150 (paired-end 150nt) sequencing on the Illumina NovaSeq 6000 (Novogene, Sacaramento, CA, USA). For miRNA sequencing, the small RNA library was constructed using the NEB Next^^®^^ Multiplex Small RNA Library Prep Set for Illumina^^®^^ (Set 1) (NEB, Ipswich, MA, USA) following manufacturer’s instructions. Subsequently, the small RNA library was sequenced on Illumina NovaSeq 6000 (Novogene, Beijing, China), and 50 bp single-end reads were generated. Raw sequence files have been deposited at NCBI’s Gene Expression Omnibus (Accession code: https://www.ncbi.nlm.nih.gov/geo/query/acc.cgi?acc=GSE190744).

### Quality control

For lncRNA, mRNA and circRNA sequencing, raw data (raw reads) of fastq format were firstly processed through in-house perl scripts. In this step, clean data were obtained by removing the following reads: (1) reads with 5′ adapter (2) reads without 3′ adapter or insert sequence (3) reads with more than 10% N (4) reads with more than 50% nucleotides with Qphred ≤20 (5) reads with ploy *A*/*T*/*G*/*C*. Adapter trimming for removing adapter sequences from the 3′ ends of reads was also performed. For miRNA sequencing, raw data of fastq format were firstly processed through custom perl and python scripts. In this step, clean data (clean reads) were obtained by removing the following reads: (1) reads with more than 10% N (2) reads with 5′ adapter contaminants (3) reads without 3′ adapter or the insert tag (4) reads with ploy *A*/*T*/*G*/*C* (5) reads with more than 30% nucleotides with Qphred ≤20. Then, high-quality clean reads were selected to do all downstream analyses.

### Mapping, assembly, and identification of transcripts

For lncRNA, mRNA sequences, the clean reads were aligned to the rat reference genome (*rattus norvegicus* genome obtained from Ensembl Rnor_6.0) with the software Hisat2 (version 2.0.5; main parameter: –no-unal -t –phred33 –rna-strandness RF –dta-cufflinks –un-conc-gz). Reads alignment results of lncRNA and mRNA were transferred to the program StringTie (version 1.3.3) for transcript assembly, and the transcripts were merged using Cuffmerge software (version 2.2.1). The current mainstream coding potential analysis method, CPC2 (version 3.2.0), Pfam (version 1.3), and CNCI, were used to predict the coding potential of the new transcripts screened of mRNA and lncRNA. And novel lncRNAs were named following the rules of the HUGO Gene Nomenclature Committee (HGNC). The small RNA tags were mapped to the reference sequence by Bowtie (version 0.12.9; main parameter: -v 0 -k 1) without mismatch to analyze their expression and distribution on the reference. Mapped small RNA tags were used to look for known miRNA. miRBase20.0 (*rattus norvegicus*) was used as reference. The circRNAs were detected and identified using find_circ and CIRI (version 2.0.5).

### Quantification and identification of differentially expressed RNA (dif-RNAs)

Quantification of the transcripts was performed using StringTie software (version 1.3.3) and Fragments Per Kilobase of transcript per Million mapped reads (FPKM) was used to determine the expression level of mRNAs and lncRNAs. miRNA and circRNA expression levels were estimated by transcript per million (TPM) through the following criteria: Normalization formula: Normalized expression = mapped readcount*1000000/Total reads. “edgeR” R package was used for differential analysis (Robinson, McCarthy & Smyth, 2010). Given the importance of small changes in RNA expression (St Laurent et al., 2013), fold changes >1.2 [up-regulated] or <1/1.2 (0.83) [down-regulated] and *p* value <0.05 were used as the threshold for differential screening to identify differential lncRNA (dif-lncRNA), mRNA (dif-mRNA), circRNA (dif-circRNA) and miRNA (dif-miRNA) (St Laurent et al., 2013; Wang et al., 2020a). The R package RNASeqPower (Hart et al., 2013) was used to retrospectively calculate the statistical power of this experiment. A power of 0.73 was found for genes that presented fold-change greater than two with a sample size of three (the number of biological replicates used for RNA sequencing).

### Spatial marker genes in the GIGR rat growth plate

To further analyze the spatial marker gene expression in the GIGR rat growth plate, we compared our dataset with previously published marker genes of R zone, P zone, and H zone of growth plate from 7-day-old SD rats (Lui et al., 2010). In this analysis, we hypothesized that the gene expression patterns are similar among the individual growth plate zones in 7-day, 21-day, and 28-day rats because the morphology of the individual zones is similar and Lui et al. (2010) have previously shown that the genes that vary with space in the rat growth plate are mostly different from those that vary with age.

### Functional enrichment analysis

Kyoto Encyclopedia of Genes and Genomes (KEGG) pathway analysis were performed using the “clusterProfiler” R package to analyze the enriched signaling pathways of differentially expressed mRNAs. Gene Ontology (GO) annotations were performed using a DAVID online tool on the screened differentially expressed genes (available online: https://david.ncifcrf.gov/) to elucidate comprehensive information on gene function by classifying genes in terms of molecular function (MF), cellular composition (CC), and biological process (BP) (Huang, Sherman & Lempicki, 2009). Then, the results were visualized with the “ggplot2” R package. False discovery rate (FDR) was calculated to correct the *p* value. The GO terms and pathways with the enriched gene count ≥2 and the significance threshold *p* < 0.05 were considered significant.

Gene set enrichment analysis (GSEA) focuses on the annotated gene set, assessing whether it is statistically significant and consistently different between two biological states (Subramanian et al., 2005). We used the “cluster profile” R package to identify significantly up- and down-regulated pathways between the Dex and control groups. All genes identified by high-throughput sequencing were used for GSEA. The significant enrichment of the gene sets followed FDR<0.25, —NES—>1.0, and nominal *p* < 0.05. A list of all genes and their membership in each GSEA gene set is in Supplementary file (Table S1).

### Prediction of miRNA Targets

We used Blastn (BLAST 2.2.28+) to filter out lncRNAs that may be miRNA precursors, then used miRanda−3.3a to predict the potential interactions between dif-miRNA and dif-lncRNA. We also used miRanda−3.3a to predict the potential interactions between dif-circRNAs and dif-miRNAs (John et al., 2004).The software miRanda−3.3a and RNAhybrid (version 2.0) were used to predict miRNA-mRNA target interactions and take intersections for subsequent analysis.

### Prediction of lncRNA Targets

Target gene prediction of lncRNAs was carried out in two ways: the cis-acting target gene prediction, and trans-acting target gene prediction (Chen, 2016). Based on the theory of cis-acting regulatory element, the protein-coding genes located within 100kb from lncRNA were selected as potential cis-acting target. While for trans-acting target prediction, the Pearson’ correlations coefficients between the coding genes and lncRNAs were calculated (—r—>0.95) and analyzed for the identification of trans-acting regulatory elements.

### ceRNA network construction

Thomson and Dinger’s ceRNA hypothesis notes that specific RNAs (such as, lncRNAs and circRNAs), which communicate and regulate by competitively binding to the RNA-induced silencing complex (RISC), can impair miRNA activity through sequestration and thereby regulating miRNA target gene expression (Thomson & Dinger, 2016). In our study, the network was constructed with the following steps: (a) Obtention of the differential expressed mRNAs, miRNAs, lncRNAs, and circRNAs by high-throughput RNA sequencing. (b) Prediction of the dif-miRNA targets to obtain dif-miRNA-dif-mRNA, dif-miRNA-dif-lncRNA and dif-miRNA-dif-circRNA regulatory relationships; Prediction of the lncRNA targets to obtain lncRNA-mRNA regulatory relationships. (c) miRNAs that are regulated by both lncRNA and mRNA were selected to construct dif-lncRNA-dif-miRNA-dif-mRNA regulatory relationships; miRNAs that are regulated by both circRNA and mRNA were selected to construct dif-circRNA-dif-miRNA-dif-mRNA regulatory relationships. (d) Integrating difference expression relationships with lncRNA-mRNA regulatory relationships (for lncRNA). (e) Further filtering of up- and down-regulated relationship pairs. (f) Integrate circRNA-miRNA-mRNA and lncRNA-miRNA-mRNA networks. Then we used Cytoscape software (version 3.7.2) to visualize the results (Shannon et al., 2003).

### Real-time quantitative fluorescence PCR (RT-qPCR)

We used TRIzol reagent according to the manufacturer’s instructions to extract total RNA from growth plates. For mRNA, PrimeScript™ RT reagent Kit with gDNA Eraser (Takara, San Jose, CA, USA) was used to convert total RNA to cDNA (37 °C for 15 min and 85 °C for 5 s) and TB Green^®^ Premix Ex Taq™ (Takara, San Jose, CA, USA) was used to amplify the cDNA as follows: 95 °C for 30 s, 40 cycles of 95 °C for 5 s and 60 °C for 34 s. For miRNA, cDNA was synthesized using the miRNA 1st strand cDNA synthesis kit (Accurate Biotechnology Co., Ltd, Hunan, China). SYBR^^®^^ Green Premix *Pro Taq* HS qPCR Kit (Accurate Biotechnology Co., Ltd, Hunan, China) was used for the RT-qPCR assay as follows: 95 °C for 30 s, 40 cycles of 95 °C for 5 s and 60 °C for 20 s. Data were normalized to GAPDH (for mRNA) and U6 (for miRNA). The primers used in these experiments are listed in Table S2 . RT-qPCR results were calculated using the 2^−ΔΔCT^ method (Livak & Schmittgen, 2001). All groups had three independent samples and all of the samples were conducted in triplicate.

### Statistical analyses

Changes in body weight, naso-anal length, bone length, height of each zone of the growth plate, percentage of positive cells were expressed as means ± standard deviation, and analyzed using SPSS 23.0 (IBM, United States) and the GraphPad Prism 6 (GraphPad, CA, United States). Differences between groups were analyzed using Student’s *t*-test. In all analyses, *p* < 0.05 was considered statistically significant.

## Results

### Establishment of the GIGR rat model

**Figure 1:**
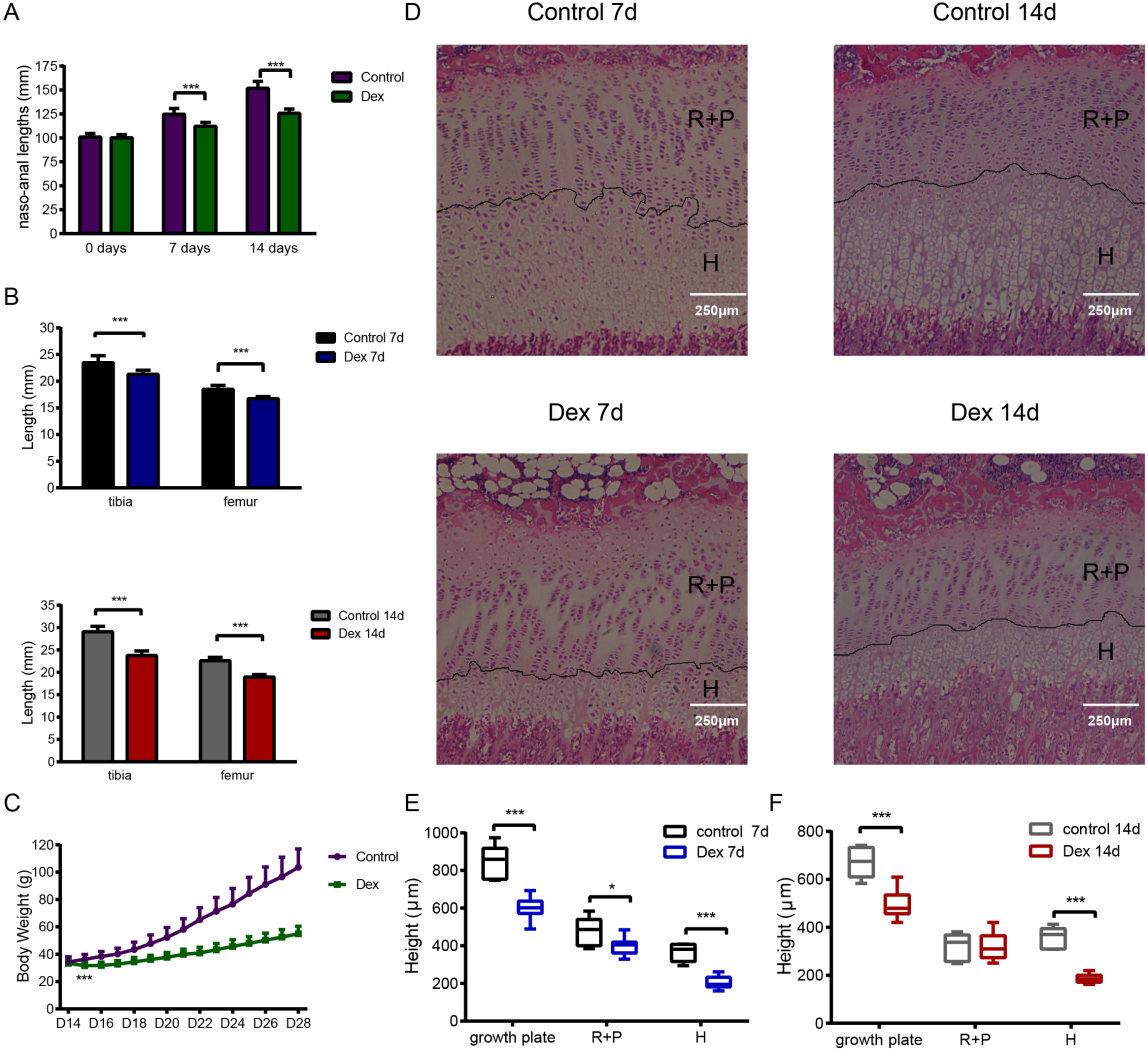
The effect of Dex administration on SD rats. Two-week-old male SD rats were treated with Dex (2.0 mg/kg body weight/d, i.p.) or saline for 7 or 14 d (*n* = 9, 7, 10, 6; Dex 7 d, Control 7 d, Dex 14 d, Control 14 d). (A) Naso-anal lengths were measured weekly. (B) Tibia and femur bone lengths were measured using Vernier calipers. (C) Growth curves tracking body weight in the control and Dex groups. (D) Histological images of HE stained growth plates from each group. Original magnification ×40. 7 (E) and 14 (F) d group growth plate widths, R+P zones (resting + proliferative zones) and H zones (hypertrophic zones) were analyzed in histologic sections from tibia growth plates (*n* = 9, 7, 10, 5; Dex 7d, Control 7d, Dex 14d, Control 14d). *: *p* < 0.05 **: *p* < 0.01 ***: *p* < 0.001 compared with the control group.

We established the rat model of GIGR *via* Dex administration (2.0 mg/kg/d, intraperitoneal (i.p.) injection). After seven days treated, the naso-anal length significantly differed between the Dex and the control groups (Fig. 1A; Dex *vs.* Control: 112.02 ± 4.15 mm *vs.* 124.57 ± 6.17 mm, *p* < 0.001, *df* = 30, *n* = 19, 13). This difference became even more significant (Fig. 1A; Dex *vs.* Control: 125.70 ± 4.41 mm *vs.* 151.74 ± 7.22 mm, *p* < 0.001, *df* = 14 *n* = 10, 6) after 14 days of Dex treatment. Similarly, after 7 days of Dex administration, compared to the control group, the lengths of tibia and femur decreased by 9.24% and 9.41%, respectively (Fig. 1B; Dex 7d *vs.* Control 7d: 16.75 ± 0.38 mm *vs.* 18.49 ± 0.75 mm, *p* < 0.001, *df* = 8, *n* = 9, 7). Dex treatment for 14 days also reduced tibia and femur length by 18.31% and 16.03% (Fig. 1B; Dex 14d *vs.* Control 14d: 18.96 ± 0.55 mm *vs.* 22.58 ± 0.76 mm, *p* < 0.001, *df* = 14, *n* = 10, 6), respectively, compared with the control group. In addition, on the second day of Dex treatment, there was a significant difference in body weight between the two groups (Fig. 1C; Dex *vs.* Control: 31.63 ± 2.57 g vs.36.32 ± 3.53 g, *p* < 0.001, *df* = 30, *n* = 19, 13).

Histological analysis of tibia growth plates was carried out in four groups of rats. The height of the growth plate, resting+proliferative (R+P) zone, and hypertrophic (H) zone in the Dex 7d group were significantly reduced compared to those of the Control 7d group (Figs. 1D, 1E; growth plate: 600.15 ± 58.70 µm *vs* 848.15 ± 90.45 µm, *p* < 0.001, *df* = 14, Dex 7d *vs.* Control 7d, *n* = 9, 7; R+P: 398.85 ± 45.48 µm *vs* 482.17 ± 71.68µm, *p* = 0.013, *df* = 14, Dex 7d *vs.* Control 7d, *n* = 9, 7; H: 204.97 ± 33.50 µm *vs* 367.90 ± 44.82 µm, *p* < 0.001, *df* = 14, Dex 7d *vs.* Control 7d, *n* = 9, 7). After 14 days of Dex treatment, the height of the growth plate and H zone in the Dex group was significantly lower than those in the control group (Figs. 1D, 1F; growth plate: 497.89 ± 63.60 µm *vs* 672.38 ± 64.56 µm, *p* < 0.001, *df* = 13, Dex 14d *vs.* Control 14d, *n* = 10, 5; H: 186.81 ± 18.05 µm *vs* 356.02 ± 45.46 µm, *p* < 0.001, *df* = 5, Dex 14d *vs.* Control 14d, *n* = 10, 5), but there was no difference in R+P zone height (Fig. 1F; R+P: 323.20 ± 56.12 µm *vs* 317.96 ± 57.33 µm, *p* = 0.87, *df* = 13, Dex 14d *vs.* Control 14d, *n* = 10, 5).

### Chondrocyte apoptosis increased and proliferation decreased upon Dex treatment

**Figure 2:**
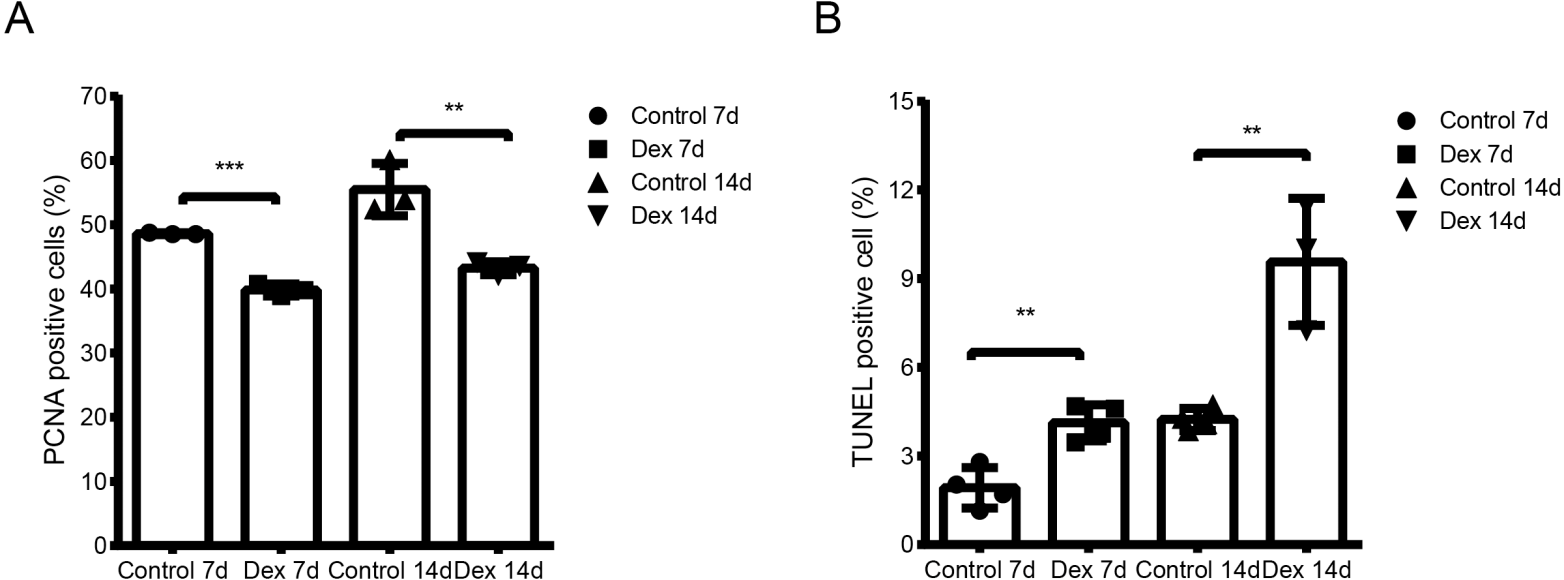
Dex effects on proliferation and apoptosis in growth plates in the GIGR rat model. (A) Quantitative TUNEL analysis in the H zone of the growth plate (n = 3–4/group). (B) PCNA analysis for cell proliferation in the P zone of the growth plate (n = 3/group). **: *p* < 0.01 ***: *p* < 0.001 compared with the control group.

We used the TUNEL assay to determine chondrocyte apoptosis rates in the hypertrophic area of the growth plate. The apoptosis rate of the Dex group was significantly higher than that of the control group (Fig. 2A, Fig. S1; Dex 7d *vs.* Control 7d: 4.13 ± 0.61% *vs.* 1.93 ± 0.69%, *p* = 0.003, *df* = 6, *n* = 4; Dex 14d *vs.* Control 14d: 9.63 ± 2.05% *vs.* 4.24 ± 0.37%, *p* = 0.003, *df* = 5, *n* = 4, 3). Results of immunohistochemistry to determine proliferating cell nuclear antigen (PCNA) expression in the P zone indicate that PCNA positivity chondrocytes, which reflect active cell division, were reduced in Dex-treated rats (Fig. 2B, Fig. S1; Dex 7d *vs.* Control 7d: 39.83 ± 0.94% *vs.* 48.60 ± 0.14%, *p* < 0.001, *df* = 4, *n* = 3; Dex 14d *vs.* Control 14d: 43.24 ± 1.02% *vs.* 55.48 ± 4.08%, *p* = 0.007, *df* = 4, *n* = 3).

All the results above indicated that the GIGR rat model had been successfully constructed, providing a firm foundation for the sample preparation of high-throughput sequencing.

### Transcriptome analysis of the GIGR model rat growth plates by high-throughput sequencing

To understand how GC alters transcriptomic profiles of whole growth plates, we collected tissues from growth plates in the proximal tibia and used high-throughput sequencing technology to analyze mRNAs, lncRNAs, circRNAs, and miRNAs expression patterns among different groups (three biological replicates per condition). Sequencing quality control data are presented in Table S3. A total of 22,462 mRNAs, 11,898 lncRNAs, 4,651 circRNAs, and 779 miRNAs were successfully mapped and identified.

**Figure 3:**
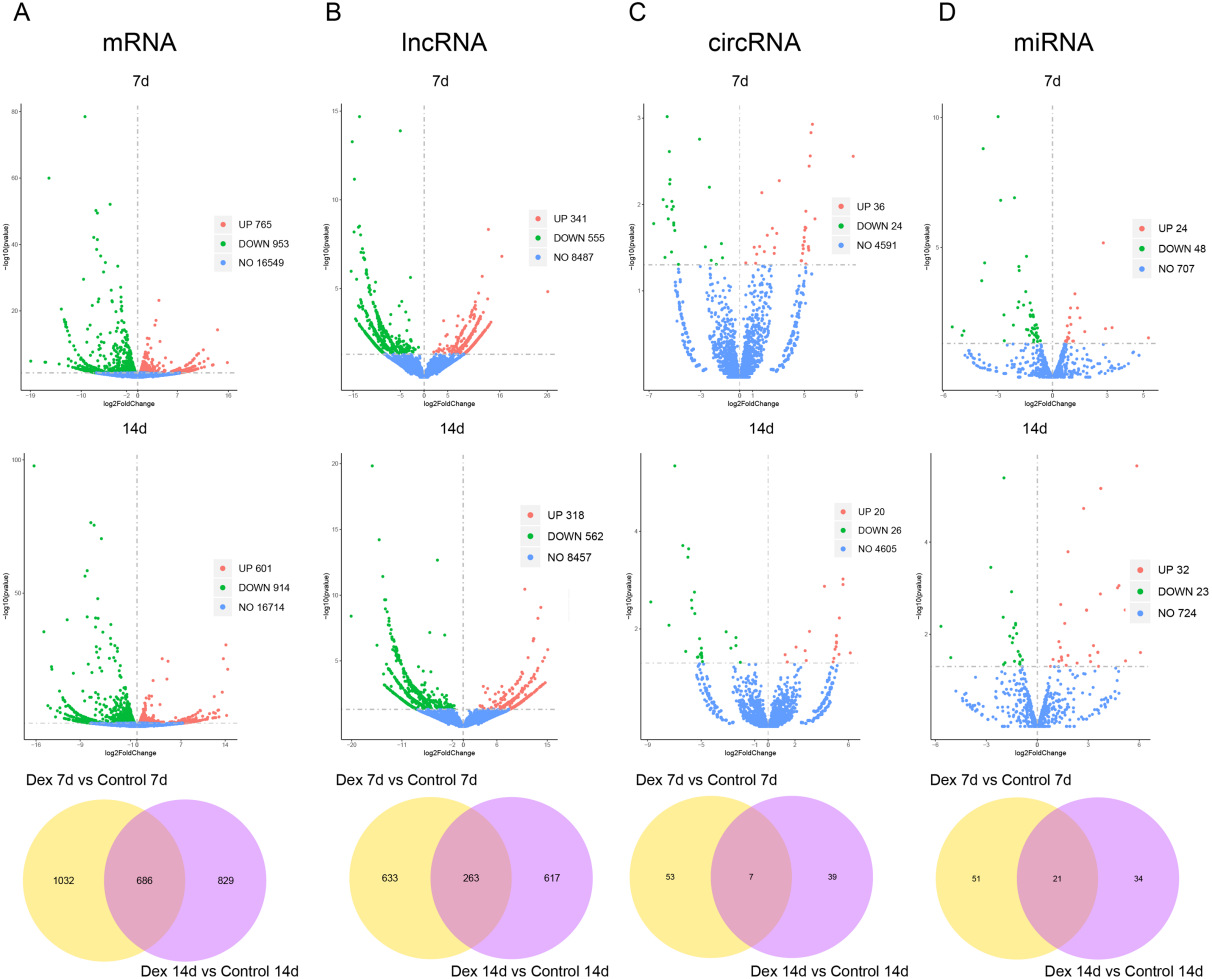
Dif-RNAs in growth plates of GIGR model rats. Volcano plots and Venn diagrams of dif-mRNAs (A), dif-lncRNAs (B), dif-circRNAs (C) and dif-miRNAs (D) of Dex group *vs.* control group. Red dots indicate up-regulated dif-RNAs and green dots indicate down-regulated dif-RNAs. Blue points refer to non-differentially expressed RNAs.

Based on the established screening criteria, 1,718 (1701 known mRNAs and 17 novel mRNAs) differentially expressed mRNAs (dif-mRNAs) were identified in the Dex 7d group (Dex 7d group *vs.* Control 7d group), of which 765 were up-regulated, and 953 were down-regulated. In the Dex 14d group (Dex 14d group *vs.* Control 14d group), 1,515 (1497 known mRNAs and 22 novel mRNAs) dif-mRNAs were identified: 601 were up-regulated and 914 were down-regulated. Six hundred and eighty-six dif-mRNAs were present in common in the Dex 7d group and the Dex 14d group (Fig. 3A, Table S4). In the Dex 7d group, 896 (138 known lncRNAs and 758 novel lncRNAs) differentially expressed lncRNAs (dif-lncRNAs) were identified, of which 341 were up-regulated and 555 were down-regulated. In the Dex 14d group, 880 (140 known lncRNAs and 740 novel lncRNAs) dif-lncRNAs were discovered, of which 318 were up-regulated and 562 were down-regulated. Two hundred and sixty-three dif-lncRNAs were found in common in the Dex 7d group and the Dex 14d group (Fig. 3B, Table S4). Sixty differentially expressed circRNAs (dif-circRNAs) were detected in the Dex 7d group. Of these, 36 were up-regulated and 24 were down-regulated. Forty-six dif-circRNAs were found in the Dex 14d group. Of these, 20 were up-regulated and 26 were down-regulated. Seven dif-circRNAs were shared by the Dex 7d group and the Dex 14d group (Fig. 3C, Table S4). Seventy-two (68 known miRNAs and 4 novel miRNAs) differentially expressed miRNAs (dif-miRNAs) were recognized in the Dex 7d group, of which 24 were up-regulated and 48 were down-regulated. Fifty-five (53 known miRNAs and 2 novel miRNAs) dif-miRNAs were identified in the Dex 14d group, of which 32 were up-regulated and 23 were down-regulated. Twenty-one dif-miRNAs were shared by the Dex 7d and the Dex 14d groups (Fig. 3D, Table S4).

We subsequently assessed expression levels of known growth plate zonal marker genes (Lui et al., 2010) in GIGR rat growth plate (dif-mRNAs shared by the Dex 7d and the Dex 14d groups). 3 out of 32 (9.38%) R zone marker genes were upregulated in Dex group compared to Control group (Table S5). None of the six P zone marker genes were differentially expressed. 14 out of 117 (11.97%) H zone marker genes were significantly different expression in Dex group compared to Control group (Table S5).

### Verification of differentially expressed mRNA in the GIGR model rats

**Figure 4:**
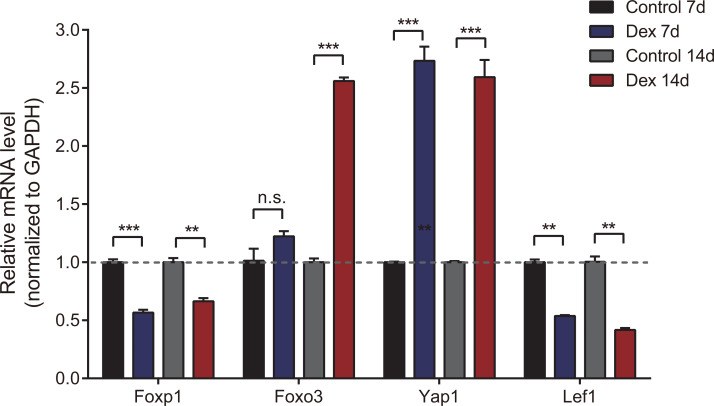
Validation of 4 dif-mRNAs by RT-qPCR. RT-qPCR was carried out to assess relative dif-mRNA expression of Foxp1, Foxo3, Yap1, and Lef1. Data are presented as means ± SEM (n = 3/group). **: *p* < 0.01 ***: *p* < 0.001 n.s.: no significance. Compared with the control group. Expression was normalized to GAPDH for mRNAs as internal controls.

To verify the accuracy of high-throughput sequencing, we performed real-time quantitative fluorescence PCR (RT-qPCR) on four differentially expressed genes (two up-regulated and two down-regulated), which were identified as transcription factors that could broadly regulate gene expression, including Foxp1, Foxo3, Yap1 and Lef1. As shown in Fig. 4, Yap1 was confirmed to be up-regulated in seven and 14d Dex-treated growth plates, while Foxp1 and Lef1 were down-regulated. Foxo3 was up-regulated in the Dex 14d group compared with the Control 14d group. The RT-qPCR results were generally consistent with high-throughput sequencing results.

### Function enrichment analyses of identified dif-mRNAs

**Figure 5:**
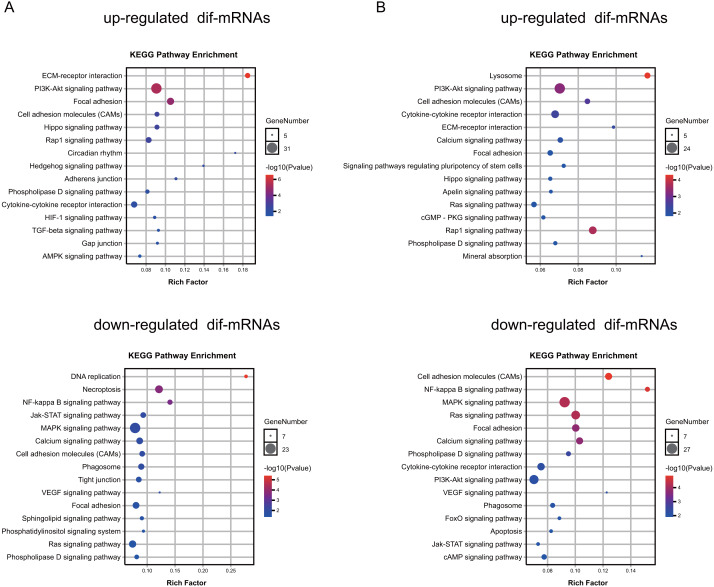
KEGG pathway analysis of dif-mRNAs. (A) Pathways enriched by up-regulated or down-regulated dif-mRNAs in the 7 d group. Dex 7 d group *vs.* Control 7 d group. (B) Pathways enriched by up-regulated or down-regulated dif-mRNAs in the 14 d group. Dex 14 d group *vs.* Control 14 d group.

To explore the function of the differentially expressed mRNAs, we identified enriched KEGG pathways and GO terms in different groups (Tables S5–S6, respectively). The results indicated that the genes up-regulated in the Dex-treated growth plates (both seven and 14 days) were associated with the PI3K-Akt signaling pathway, and Hippo signaling pathway (Fig. 5A, Table S6). On the other hand, down-regulated genes in the Dex-treated growth plates (both seven and 14 days) mainly consisted of the pathways, such as the MAPK signaling pathway, NF-kappa B signaling pathway, and Calcium signaling pathway (Fig. 5B, Table S6).

Moreover, up-regulated genes in Dex group (both seven and 14 days) mainly consisted of the GO terms, such as the extracellular matrix (GO:0031012), angiogenesis (GO:0001525), ossification (GO:0001503), calcium ion binding (GO:0005509), transforming growth factor beta binding (GO:0050431), chondrocyte differentiation (GO:0002062), bone development (GO:0060348), negative regulation of cell proliferation (GO:0008285), response to glucocorticoid (GO:0051384), and negative regulation of extrinsic apoptotic signaling pathway (GO:2001237) (Table S7). Down-regulated genes in Dex group (both seven and 14 days) mainly consisted of the GO terms, including adaptive immune response (GO: 0002250), cytoplasm (GO:0005737), intracellular signal transduction (GO:0035556), phosphotyrosine binding (GO:0001784), protein autophosphorylation (GO:0046777), regulation of cell shape (GO:0008360), positive regulation of transcription, apoptotic process (GO:0006915), regulation of cell size (GO:0008361), positive regulation of angiogenesis (GO:0045766), and calcium channel regulator activity (GO:0005246) (Table S7), which are closely related to the cellular proliferation, apoptosis, differentiation, the maturation of growth plate, bone metabolism, and immune response.

**Figure 6:**
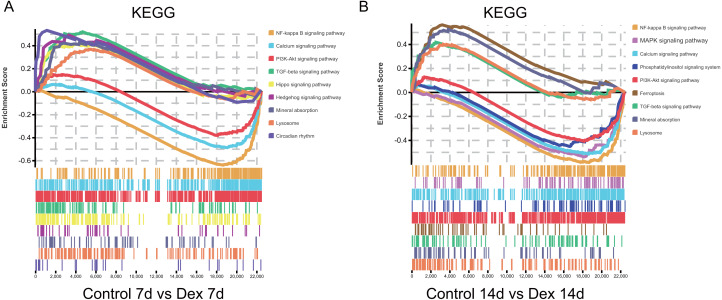
GSEA analysis of mRNA (selected results). (A) Pathways enriched in the 7 d group (selected results). (B) Pathways enriched in the 14 d group (selected results). Only gene sets with NES >1.0, *p* < 0.05 and FDR *q* < 0.25 were considered significant.

Furthermore, we found that some pathways were significantly enriched in both up- and down-regulated genes. To clarify whether these pathways were activated or suppressed after Dex treatment we performed GSEA analysis (—NES—>1.0, FDR<0.25, nominal *p* < 0.05) (Table S1). The GSEA analysis showed that the TGF-*β* signaling pathway, Hippo signaling pathway, Circadian rhythm, Hedgehog signaling pathway, mineral absorption, and lysosome were significantly activated after seven days of Dex treatment. With the prolonged Dex treatment (up to 14 days), the ferroptosis, mineral absorption, lysosome, and TGF-*β* signaling pathway were significantly activated (Fig. 6A, Table 1). On the other hand, the NF-kappa B signaling pathway, Calcium signaling pathway, and PI3K-Akt signaling pathway were suppressed after 7 days of Dex treatment. With the prolonged Dex treatment (up to 14 days), the MAPK signaling pathway and Phosphatidylinositol signaling system exhibited additional suppressed (Fig. 6B, Table 1). These pathways were highly related to the chondrocyte development and cartilage homeostasis.

**Table 1:** GSEA analysis of mRNA (selected results). Gene sets with NES>1.0, *p* < 0.05 and FDR *q* < 0.25 were considered significant.

1. The detailed information of GSEA in the Dex 7d group (selected results).				
Gene set name	ES	NES	*p*-Value	FDR *q*-value
TGF-beta signaling pathway	0.519	1.819	0.000	0.060
Hippo signaling pathway	0.410	1.547	0.003	0.096
Circadian rhythm	0.531	1.513	0.026	0.103
Hedgehog signaling pathway	0.491	1.509	0.022	0.095
Mineral absorption	0.424	1.378	0.045	0.192
Lysosome	0.367	1.356	0.011	0.206
NF-kappa B signaling pathway	−0.643	−2.363	0.000	0.000
Calcium signaling pathway	−0.491	−1.842	0.000	0.001
PI3K-Akt signaling pathway	−0.383	−1.477	0.000	0.043

2. The detailed information of GSEA in the 14d group (selected results).				
Gene set name	ES	NES	*p*-Value	FDR *q*-value
Ferroptosis	0.562	1.884	0.000	0.030
Mineral absorption	0.524	1.791	0.000	0.042
Lysosome	0.399	1.549	0.000	0.101
TGF-beta signaling pathway	0.417	1.510	0.000	0.120
NF-kappa B signaling pathway	−0.589	−1.853	0.000	0.000
Calcium signaling pathway	−0.518	−1.657	0.000	0.006
Phosphatidylinositol signaling system	−0.547	−1.598	0.001	0.015
MAPK signaling pathway - fly	−0.461	−1.337	0.039	0.197
PI3K-Akt signaling pathway	−0.409	−1.329	0.002	0.209

### ceRNA Network Construction

Based on the ceRNA hypothesis, lncRNAs/circRNAs could modulate gene expression as miRNA sponges (Thomson & Dinger, 2016). More specifically, circRNAs, lncRNAs, and mRNAs, as ceRNAs, can impair miRNA activity by competitive binding to miRNA response elements (MREs), thereby upregulating miRNA target gene expression. Therefore, the expression level of ceRNA should be negatively correlated with miRNA expression and positively correlated with mRNA expression. We constructed the up-regulated and down-regulated ceRNA networks based on the predicted relationships and the corresponding expression data to obtain more reliable results. In total, 291 nodes and 453 edges were finally obtained for the ceRNA network associated with the Dex 7d group, including 25 up-regulated lncRNAs and 55 down-regulated lncRNAs, five up-regulated circRNAs and eight down-regulated circRNAs, 12 up-regulated miRNAs and 17 down-regulated miRNAs, as well as 78 up-regulated mRNAs and 91 down-regulated mRNAs (Fig. S2, Table S8). For the ceRNA network associated with the Dex 14d group, there were 153 nodes and 245 edges, including 16 up-regulated lncRNAs and 56 down-regulated lncRNAs, four up-regulated circRNAs and four down-regulated circRNAs, nine up-regulated miRNAs and nine down-regulated miRNAs, as well as 16 up-regulated mRNAs and 34 down-regulated mRNAs (Fig. S2, Table S8).

To explore the functions of the ceRNA network, dif-mRNAs of the ceRNA network were analyzed by KEGG analysis. The KEGG analysis results indicated that the most significantly enriched pathways of the dif-mRNAs in the Dex 7d group ceRNA network were Metabolic pathways, MAPK signaling pathway, Rap1 signaling pathway, Pathways in cancer, and PI3K-Akt signaling pathway (Table S9). The most significantly enriched pathways of dif-mRNAs in the Dex 14d group ceRNA network were MAPK signaling pathway and Metabolic pathways (Table S9).

**Figure 7:**
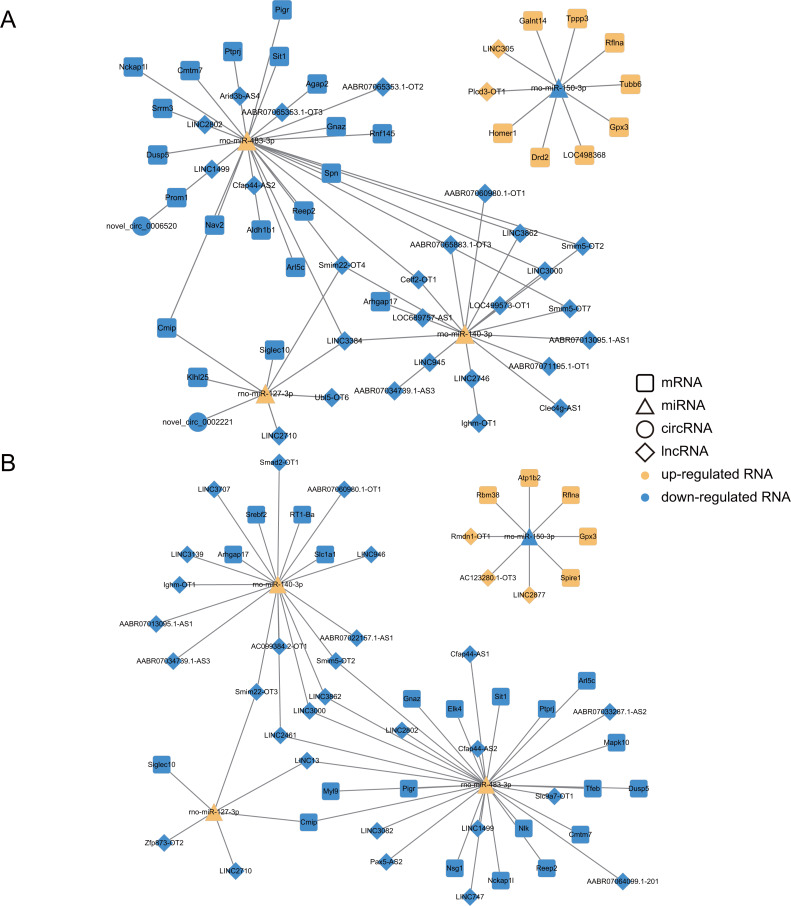
Construction of ceRNA networks associated with four miRNAs (validated by RT-qPCR). (A) Construction of the Dex 7 d group ceRNA network (about four validated miRNA). (B) Construction of the Dex 14 d group ceRNA network (about 4 validated miRNA). The four RT-qPCR validated miRNAs included miR-483-3p, miR-127-3p, miR-140-3p and miR-150-3p. The rounded rectangles indicate mRNAs, triangles represent miRNAs, prisms represent lncRNAs, and circles indicate circRNAs. The yellow indicates the up-regulated RNAs. The blue indicates the down-regulated RNAs.

To further discover networks essential nodes, the selected lncRNA, circRNAs, miRNAs and mRNAs were examined with regard to degree of the nodes. It was actually found that the degrees of the miRNA nodes were higher than those of the lncRNA, circRNA and mRNAs, suggesting that miRNAs accounted for the major percentage of the ceRNA network (Table S8). Meanwhile, four up-regulated miRNAs (miR-483-3p, miR-127-3p, miR-140-3p and miR-204-5p) and four down-regulated miRNAs (miR-150-3p, miR-20b-3p, miR-449a-5p and miR-17-2-3p) were found to be expressed in the same trend across the Dex 7d group and the Dex 14d group ceRNA network, suggesting that they might participate in vital functions in the GIGR ceRNA network. We selected four (miR-483-3p, miR-127-3p, miR-140-3p and miR-150-3p) of them for RT-qPCR validation (Fig. S3). The expression patterns of these miRNAs were consistent with the results obtained by high-throughput sequencing. Based on the validation results of RT-qPCR, miR-483-3p, miR-127-3p, miR-140-3p, miR-150-3p and its predicted targets were selected to further display the ceRNA network (Fig. 7). As shown in Fig. 7, there were complex regulatory relationships between RNAs. For example, in ceRNA network of the Dex 7d group, LINC2710, Ubl5-OT6, LINC3384, Smim22-OT4 and novel_circ_0002221 were predicted as ceRNAs of miR-127-3p, which targeted Cmip, Klhl25, and Siglec10 (Fig. 7A). From the Dex 14d group ceRNA network, we identified LINC13 as competing for binding to miR-127-3p and miR-483-3p, thereby affecting Cmip expression (Fig. 7B).

## Discussion

GIGR is a common adverse effect on the skeletal system of long-term GC use in children (Simon et al., 2002). The evidence of an important role of ncRNAs in the regulation of chondrocyte development continues to increase, and the association between ncRNAs and chondrocyte related disorders, such as osteoarthritis, had been investigated by several group (Ratneswaran & Kapoor, 2021). However, the role of ncRNAs in GIGR remains unclear. Recent studies have shown that the indirect and direct effects of GCs on the growth plates have been considered to be the underlying causes for GC effects on growth (Hartmann et al., 2016). Therefore, it is necessary to understand how GC alters transcriptomic profiles of growth plates. In this study, the GIGR rat model was established by Dex injection (Liu et al., 2018; Wood et al., 2018). To elucidate the pathogenesis of GIGR, high-throughput sequencing technology and bioinformatics analysis were applied to systematically analyze the differentially expressed lncRNAs, circRNAs, miRNAs and mRNAs in the growth plates of GIGR rats and the ceRNA regulatory networks were also established.

Many differentially expressed genes in the growth plate of GIGR rats were revealed in the current study. For example, we found that some growth plate spatial marker genes were significantly different between the Dex and Control groups, which were mainly focused on the R and H zone (Table S5). Unfortunately, the relevant mechanisms of action of these genes in individual zones of the growth plate are not well characterized. We speculated that the reduction in the height of the H zone of the growth plate in GIGR rats may be closely related to the changes in H zone marker genes (Table S5), however, the specific regulatory mechanisms remain to be explored.

In addition, we validated four genes (transcription factors) which may be closely related to the development of GIGR (Fig. 4). Yap1 is a key downstream effector of the Hippo pathway (Xie et al., 2020). The Hippo signaling pathway has been found to mediate chondrocyte differentiation and postnatal growth (Roselló-Díez & Joyner, 2015). According to reports, excessive Yap1 activity impairs chondrocyte maturation by acting through Runx2, leading to narrower growth plate height and shorter stature in mice (Deng et al., 2016). Nonetheless, the function of Yap1 in GIGR is still unclear. Further *in vivo* and *in vitro* studies may help investigate its role in the pathogenesis of GIGR. Foxp1 and Foxo3 belong to the Forkhead box (Fox) family. Fox family transcription factors, characterized by a highly conserved forkhead DNA-binding domain, are involved in regulating cell growth, differentiation, cell cycle, apoptosis, and metabolism (Herman, Todeschini & Veitia, 2021). Increasing evidence has shown that the Fox family also affected endochondral osteogenesis. Zhao et al. constructed Foxp1 knock-out mice and found that loss of Foxp1 in the fetal growth plate shortened the height of the H zone and reduced cellular proliferation in the P zone (Zhao et al., 2015). Furthermore, after birth, with the cumulative effect of Foxp1 deletion, the body length and limb bone length of the mice were significantly shorter compared to the control group. This suggests, at least in part, that decreased Foxp1 expression is closely associated with short stature. Eelen et al. (2016) found that knocking out the mice Foxo3 would affect skeletal development. Recently, it was found that Foxo3 and Runx1 protein co-localize in the proliferative chondrocytes of mouse proximal tibia growth plate (Yuan et al., 2022). In our study, Foxo3 expression increased after 14 days of GC administration, but did not change after 7 days of GC administration. We speculated that Foxo3 may engage in the GIGR development by interfering with chondrocyte proliferation, but this effect needs to accumulate to a certain extent to be effective. Lef1 is mainly involved in the classical Wnt/*β*-catenin signaling pathway and mediates its nuclear response (Zhan, Rindtorff & Boutros, 2017). It has been reported that Wnt signaling can interact with PTHrP (parathyroid hormone-related protein) and Ihh (Indian hedgehog) to co-regulate bone growth (Hallett, Ono & Ono, 2019). The down-regulation of Lef1 expression after GC treatment may impair the related signaling pathway. Further studies are expected to help clarify the role of these genes in the development of GIGR.

Function enrichment analyses of dif-mRNAs revealed that many pathways were affected upon Dex treatment. In particular, we found that sustained high-dose exposure to GCs can significantly suppressed pathways involved PI3K-Akt signaling pathway and NF-kappa B signaling pathway, while activated TGF-*β* signaling pathway (both in 7- and 14-day group). PI3K-Akt signaling pathway has been found to be associated with GIGR in previous studies (Chrysis et al., 2005). Studies have shown that Dex could inhibit Akt phosphorylation and induce chondrocyte apoptosis by inhibiting the PI3K-Akt signaling pathway (Chrysis et al., 2005; Hu et al., 2017). NF-kappa B signaling pathway has been reported to be involved in endochondral ossification (Kobayashi et al., 2016). Notably, the suppression of the NF-kappa B signaling pathway could increase chondrocyte apoptosis and inhibit proliferation (Jimi, Fei & Nakatomi, 2019), which is in line with our findings. TGF-*β* signaling pathway participates in regulating the chondrocyte differentiation and maturation of growth plate (Wang, Rigueur & Lyons, 2020c). Li et al. (2006) have shown Dex could decrease cell proliferation through enhancing TGF- *β*1 signaling and TGF- *β*1 receptor expression in human prostate cancer cells. Furthermore, TGF- *β* can inhibit the downstream BMP signaling pathway (Wang, Rigueur & Lyons, 2020c), which is crucial for osteogenesis (Salazar, Gamer & Rosen, 2016). Therefore, we supposed that the activated TGF- *β* signaling pathway in GIGR rats may be involved in the pathogenesis of GIGR.

We also observed that the most notable enriched biological processes of down-regulated dif-mRNAs (both in the Dex 7d group and the Dex 14d group) were immune-related terms (Table S7). which seemed to mean that the immune system plays an important role in regulating longitudinal bone growth. The interaction between the immune and skeletal systems has long been recognized. The skeletal and immune systems share a variety of different cytokines and signaling molecules and thus influence each other (Okamoto & Takayanagi, 2019). But the literature provides little information about the roles of these immune-related differentially expressed genes in the GIGR. Some immunodeficiency diseases, such as adenosine deaminase (ADA) deficiency (Scott et al., 2017), DiGeorge syndrome (Kobrynski & Sullivan, 2007), and STAT5b deficiency (Bernasconi et al., 2006) manifested as the abnormal development of cartilage. Athymic mice, with spontaneously mutated T lymphocyte immunodeficiency, have also shown shortened tibia bone length (McCauley et al., 1989). Reportedly, growth hormone insensitivity caused by immune dysfunction can lead to growth retardation (Bernasconi et al., 2006). This may partially explain the possible link between the use of GCs and the down-regulation of immune-related terms.

We also analyzed the expression levels of lncRNAs, circRNAs, miRNAs and mRNAs in the growth plates of rats in the 7d and 14d groups of the GIGR model and identified ceRNA regulatory networks, which revealed potential ceRNA regulation mechanisms in GIGR. In our study, miRNAs were identified as key nodes of the ceRNA network (Table S8). Indeed, miRNAs have been shown to play an important regulatory role in the growth plate (Nakamichi et al., 2020). Studies have shown that miR-140 (including miR-140-3p and miR-140-5p) is one of the miRNAs specifically and highly expressed in the growth plate, which plays a role in regulating the chondrogenesis (Woods et al., 2020). Deletion of miR-140 in mice resulted in a mild skeletal defect with shortening of limbs (Miyaki et al., 2010). However, most studies have focused on miR-140-5p rather than miR-140-3p. In our experiments, exposure to Dex resulted in down-regulation of miR-140-3p expression in rat growth plates (Fig. S3). In addition, in the ceRNA network (both in 7d group and 14d group) we predicted that lncRNA, including smim5-OT2, LINC3000, AABR07013095.1-AS1, LINC3862, AABR07034739.1-AS3, Ighm-OT1 and AABR07060980.1-OT1, could sponge miR-140-3p to regulate the expression of Arhgap17 (Fig. 7A). Arhgap17 is a RhoGTPase activating protein involved in the maintenance of tight junction and vesicle trafficking (Lee et al., 2016). Recently, it has been reported that Arhgap17 has an anti-apoptosis function (Wang et al., 2020b). In our study, we predicted that the lncRNA-miR-140-3p-Arhgap17 axis may participate in biological pathways related to growth retardation. We also predicted that LINC2710 could sponge miR-127-3p to regulate the expression of Cmip and Siglec10, which was identified both in 7d group and 14d group (Fig. 7). miR-127-3p has been found to be a cancer suppressor in osteosarcoma, and overexpression of miR-127-3p inhibited proliferation and stimulated apoptosis of osteosarcoma cells (Wang et al., 2018). Zhang et al. (2017) found that knocking down Cmip can affect cell survival by affecting MAPK signaling pathway delivery. Previous reports and our findings suggested that miR-127-3p could be the potent regulator of the GIGR. However, these predicted results require further experimental validation. In addition, we found that the predictions of the ceRNA network for biological pathways were largely consistent with the predictions of our mRNA data (*e.g.*, at the level of PI3K-Akt signaling pathway, MAPK signaling pathway and Metabolic pathways). These results suggest that the ceRNA networks we predicted in this paper might be strongly associated with the pathogenesis of GIGR.

It should be noted that the present study has some limitations. Firstly, the relatively limited number of high-throughput sequencing rats has more or less influenced the analysis of experimental results. Moreover, although we established the ceRNA regulatory network based on bioinformatics prediction and found some changes in biological pathways after Dex treatment, systematic functional experiments are still needed to further determine their role in GIGR. Furthermore, many novel lncRNAs and circRNAs were identified in our study, whereas little annotation information is available about these ncRNAs. The biological functions of these ncRNAs in endochondral ossification need to be further explored.

## Conclusions

Our study detected and analyzed mRNA, lncRNA, circRNA, and miRNA changes in the growth plates in the rat model of GIGR and revealed novel connections between differentially expressed RNAs and the pathogenesis of GIGR. Bioinformatics analyses identified several molecules and signaling pathways that may contribute to GIGR. These results can lay the foundation for analysis of the molecular mechanisms of GIGR. However, further functional characterization validation is required to delineate exact mechanistic details.

##  Supplemental Information

10.7717/peerj.14603/supp-1 Supplemental Information 1 Detailed information about GSEA analysis

10.7717/peerj.14603/supp-2 Supplemental Information 2 Primer sequences

10.7717/peerj.14603/supp-3 Supplemental Information 3 Total reads and mapping ratio information for control and Dex groups

10.7717/peerj.14603/supp-4 Supplemental Information 4 Detailed information on differentially expressed mRNAs, lncRNAs, miRNAs and circRNAs

10.7717/peerj.14603/supp-5 Supplemental Information 5 The differential expression of spatial marker genes in the GIGR rat growth plate

10.7717/peerj.14603/supp-6 Supplemental Information 6 KEGG analysis of dif-mRNAs

10.7717/peerj.14603/supp-7 Supplemental Information 7 Gene Ontology analysis of dif-mRNAs

10.7717/peerj.14603/supp-8 Supplemental Information 8 The details information of the ceRNA networks associated with the Dex 7d group and the Dex 14d group

10.7717/peerj.14603/supp-9 Supplemental Information 9 The pathways of mRNA enrichment in the Dex 7d group or Dex 14d group ceRNA network mRNAs in Fig. S2(Table S8) were used for KEGG enrichment analysis.

10.7717/peerj.14603/supp-10 Supplemental Information 10 Representative images of fluorescent TUNEL analysis and immunohistochemical staining for PCNA in rat growth plates (A) Representative images of fluorescent TUNEL analysis in rat growth plates. Apoptotic cells are stained green and nuclei are stained blue (DAPI). Original magnification ×200. (B) Representative images of immunohistochemical staining for PCNA in rat growth plates . Original magnification ×400.

10.7717/peerj.14603/supp-11 Supplemental Information 11 Construction of ceRNA networks associated with the Dex 7d group and the Dex 14d group (A) Construction of the Dex 7d group ceRNA network. (B) Construction of the Dex 14d group ceRNA network. The rounded rectangles indicate mRNAs, triangles represent miRNA, prisms represent lncRNAs, and circles indicate circRNAs. The yellow indicates the up-regulated RNAs. The blue indicates the down-regulated RNAs.

10.7717/peerj.14603/supp-12 Supplemental Information 12 Validation of 4 dif-miRNAs by RT-qPCR RT-qPCR was carried out to assess relative dif-miRNA expression of miR-483-3p, miR-127-3p, miR-140-3p and miR-150-3p . Data are presented as means ± SEM (n =3/group). **: *p* < 0.01 ***: *p* < 0.001 n.s.: no significance. Compared with the control group. Expression was normalized to U6 for miRNAs as internal controls.

10.7717/peerj.14603/supp-13 Supplemental Information 13 Raw data of Figs. 1, 2, 4 and Fig. S3

10.7717/peerj.14603/supp-14 Supplemental Information 14 Author Checklist - Full

## Additional Information and Declarations

### Data Availability

The following information was supplied regarding data availability: The datasets generated and analysed during the current study are available in the GEO repository: GSE190744.
