# Supplementary material for: Transcription analyses of differentially expressed mRNAs, lncRNAs, circRNAs, and miRNAs in the growth plate of rats with glucocorticoid-induced growth retardation
Source: PeerJ. 2023 Jan 16;11:e14603. doi: 10.7717/peerj.14603 (PMC9851049; doi:10.7717/peerj.14603)
Supplement: Supplemental Information 2 [file peerj-11-14603-s002.docx]

**Table S2 Primer sequences**

| **Gene name** | **Forward Primer (5'-3')** | **Reverse Primer (5'-3')** |
| --- | --- | --- |
| *Lef1* | CACCGATGAGATGATCCCCT | GTTGCTGGCTGGGATGATTT |
| *Foxp1* | ACCCCATACACGTCAAGGAG | AATCTGGACTGTGGTTGGCT |
| *Foxo3* | GAGTCCATCATCCGTAGCGA | TTCCCCACGTTCAAACCAAC |
| *Yap1* | CATGCTCTCCCAACTGAACG | CATCCTGAGTCATGGCTTGC |
| *GAPDH* | GGCACAGTCAAGGCTGAGAATG | ATGGTGGTGAAGACGCCAGTA |
| *miR-483-3p* | CTCCTCCCCTCCCGTCTT |  |
| *miR-127-3p* | GATCCGTCTGAGCTTGGCA |  |
| *miR-140-3p* | CCACAGGGTAGAACCACGG |  |
| *miR-150-3p* | GCGTGGCCTGGGGGAAAA |  |
| *U6* | GGAACGATACAGAGAAGATTAGC | TGGAACGCTTCACGAATTTGCG |
|  |  |  |
