# Supplementary material for: Transcription analyses of differentially expressed mRNAs, lncRNAs, circRNAs, and miRNAs in the growth plate of rats with glucocorticoid-induced growth retardation
Source: PeerJ. 2023 Jan 16;11:e14603. doi: 10.7717/peerj.14603 (PMC9851049; doi:10.7717/peerj.14603)
Supplement: Supplemental Information 3 [file peerj-11-14603-s003.docx]

**Table S3 Total reads and mapping ratio information for control and Dex groups**

For the chain-specific library

| **Sample** | **Total raw reads (G)** | **Clean reads Q30 (%)** | **GC content (%)** | **Total mapping ratio (%)** |
| --- | --- | --- | --- | --- |
| c_7d_1 | 14.38 | 94.83 | 52.62 | 95.81 |
| c_7d_2 | 12.8 | 94.69 | 53.79 | 95.9 |
| c_7d_3 | 16.15 | 95.13 | 53.53 | 96.25 |
| d_7d_1 | 15.34 | 94.64 | 52.18 | 95.66 |
| d_7d_2 | 14.83 | 94.31 | 52.77 | 95.75 |
| d_7d_3 | 14.94 | 94.89 | 53.42 | 96.06 |
| c_14d_1 | 14.13 | 93.73 | 51.33 | 95.24 |
| c_14d_2 | 14.13 | 94.81 | 52.37 | 95.96 |
| c_14d_3 | 15.43 | 94.48 | 52.81 | 95.88 |
| d_14d_1 | 14.54 | 95.08 | 52.09 | 95.5 |
| d_14d_2 | 13.79 | 94.66 | 52.86 | 95.91 |
| d_14d_3 | 14.51 | 94.2 | 53.24 | 96.02 |

For the small RNA library

| **Sample** | **Total raw reads (G)** | **Clean reads Q30 (%)** | **GC content (%)** | **Total mapping ratio (%)** |
| --- | --- | --- | --- | --- |
| c_7d_1 | 0.811 | 93.48 | 50.09 | 94.91 |
| c_7d_2 | 0.770 | 93.94 | 49.33 | 96.67 |
| c_7d_3 | 0.778 | 93.44 | 49.82 | 95.51 |
| d_7d_1 | 0.844 | 94.09 | 49.85 | 95.30 |
| d_7d_2 | 0.776 | 94.26 | 50.16 | 95.24 |
| d_7d_3 | 0.798 | 93.31 | 49.76 | 95.70 |
| c_14d_1 | 0.783 | 91.78 | 49.35 | 93.20 |
| c_14d_2 | 0.865 | 92.00 | 49.54 | 96.41 |
| c_14d_3 | 0.827 | 91.54 | 50.37 | 93.36 |
| d_14d_1 | 0.827 | 93.88 | 49.60 | 94.96 |
| d_14d_2 | 0.782 | 93.27 | 50.30 | 92.59 |
| d_14d_3 | 0.788 | 93.08 | 50.79 | 95.28 |
