# Supplementary figures and images for: Transcription analyses of differentially expressed mRNAs, lncRNAs, circRNAs, and miRNAs in the growth plate of rats with glucocorticoid-induced growth retardation
Source: PeerJ. 2023 Jan 16;11:e14603. doi: 10.7717/peerj.14603 (PMC9851049; doi:10.7717/peerj.14603)

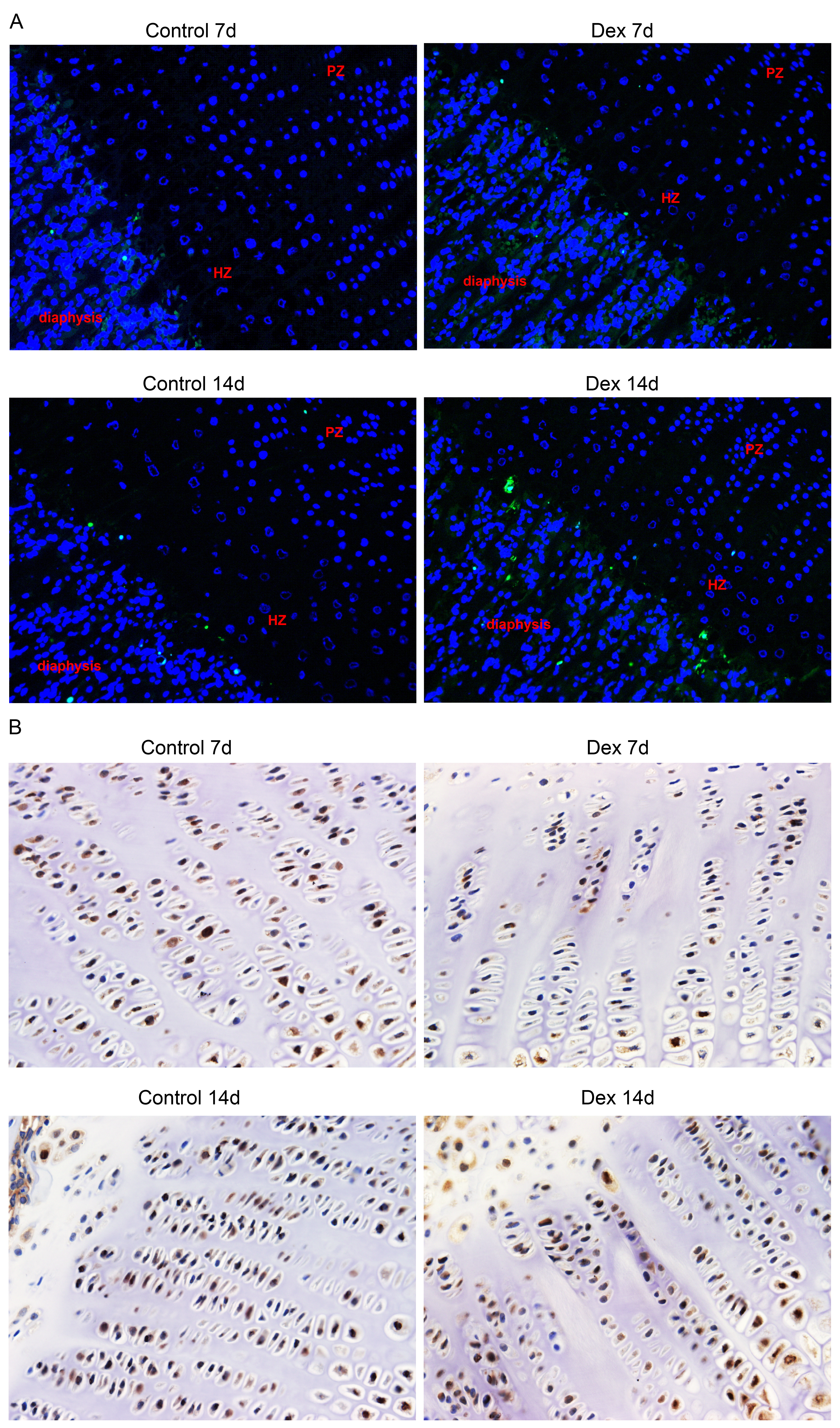

Supplement: Supplemental Information 10 — (A) Representative images of fluorescent TUNEL analysis in rat growth plates. Apoptotic cells are stained green and nuclei are stained blue (DAPI). Original magnification ×200. (B) Representative images of immunohistochemical staining for PCNA in rat growth plates . Original magnification ×400. [file peerj-11-14603-s010.png]
